# Supplementary material for: A hybrid power load forecasting model using BiStacking and TCN-GRU
Source: PLoS One. 2025 Apr 28;20(4):e0321529. doi: 10.1371/journal.pone.0321529 (PMC12036927; doi:10.1371/journal.pone.0321529)
Supplement: Attachment — Submitted filename: Response to Reviewers.docx [file pone.0321529.s002.docx]

**Response to Reviewers**

**Dear Editors and** **Reviewers:**

We sincerely thank the Editors and reviewers for their insightful and constructive comments on our manuscript, *A Hybrid Power Load Forecasting Model Combining Ensemble Learning and Deep Learning: BiStacking+TCN-GRU*. Your valuable feedback has provided essential guidance in refining the paper. We greatly appreciate the time and effort you dedicated to reviewing our work. Your comments were instrumental in identifying areas for improvement, and the revisions made in response to your suggestions have significantly enhanced the manuscript. Below is our detailed response to each of the reviewers' comments.

**Response to comments by Editors：**

Thank you for your valuable feedback. We have thoroughly reviewed the document, making the necessary revisions to enhance readability and address all minor grammatical issues. Additionally, we have ensured that data is consistently used in its correct plural form throughout the document. We have also revised the image names as per the PLOS ONE style requirements.

**Response to comments by Reviewer #1：**

**Comments：**

**1.** *How might the model's reliance on historical data influence its performance during unprecedented load variations, such as during extreme weather events*

**Response:**

Regarding the model's performance in extreme weather events, we integrated meteorological data (such as temperature and humidity) with historical load data to enhance the BiStacking+TCN-GRU model's adaptability to load fluctuations.

The model primarily handles data under normal weather conditions during routine operation. However, to address extreme weather events that may occur (such as heavy rainfall or snowfall), a model replication strategy is employed to manage these special cases. Specifically, when an extreme weather event is encountered for the first time, load and meteorological data under these conditions are manually recorded, and these data are then used to train a model replica for that specific type of weather. This approach minimizes the influence of normal data on the prediction results for extreme weather by constructing a dedicated model tailored to such extreme conditions.

When similar weather events recur, the corresponding predictive model is manually selected to perform load forecasting, thereby enhancing forecasting accuracy under extreme weather conditions. As data on extreme weather events continues to accumulate, the model replicas are iteratively updated, further improving their generalization capabilities and predictive accuracy across diverse weather scenarios.

To provide a clearer understanding of the strategies employed for addressing extreme weather events, we have elaborated on the relevant details in the conclusion.

**2.** *What specific features did the PCC algorithm identify as crucial for improving prediction accuracy in power load forecasting?*

**Response:**

We have provided a detailed description of the key features identified by the PCC algorithm in Section 4.3.2 of the revised manuscript. Our analysis indicates that temperature, humidity, and rainfall are crucial factors influencing the prediction accuracy of the electricity load forecasting model. This additional information has been incorporated into the manuscript to enhance the clarity of the factors that impact the model's performance.

**3.** *How does the performance of the BiStacking+TCN-GRU model compare to other state-of-the-art models in the literature on short-term load forecasting?*

**Response:**

We conducted additional experiments to compare our model with four leading algorithms: Transformer, Informer (DOI: 10.1609/aaai.v35i12.17325), FEDformer (DOI: 10.48550/arXiv.2201.12740), and Autoformer (DOI: arxiv-2106.13008). These models were evaluated in terms of prediction accuracy, training time, and memory usage, with the results presented in Table 2 in Section 4.3.3 of the revised manuscript. Furthermore, a comparison of our model with the most advanced models currently available is also provided in Table 1.

**Table 1.** Comparison of resource consumption of deep learning models

| **Model** | **Epochs** | **Time(s)** | **MAE** | **Memory Usage (MB)** |
| --- | --- | --- | --- | --- |
| GRU | 50 | **325.86s** | 45.5876 | **82.73** |
| FEDformer | 20 | 1882.18s | 43.0874 | 1228.83 |
| Transformer | 20 | 964.30s | 41.2293 | 1474.56 |
| TCN | 50 | 504.03s | 41.0946 | 245.76 |
| Informer | **20** | 2130.02s | 40.6356 | 2051.22 |
| TCN-GRU | 50 | 810.18s | 37.7232 | 301.48 |
| Autoformer | 20 | 1501.58s | 35.4563 | 1064.96 |
| **BiStacking+TCN-GRU** | 50 | 1046.74s | **22.5206** | 381.77 |

According to the analysis of the table data, models based on the encoder-decoder architecture require longer training times and higher computing resource requirements due to their structural complexity. In addition, models based on the encoder-decoder architecture and attention mechanisms typically have high hardware requirements during deployment and necessitate specialized training for staff in operation and maintenance, which significantly increases implementation costs. In contrast, the BiStacking+TCN-GRU model not only substantially reduces computational resource consumption and training time but also maintains high prediction accuracy. It is straightforward to operate and maintain, ensuring seamless workflow transitions and stable system performance even in the event of changes in operation or maintenance personnel. These advantages make it well-suited for rapid deployment and long-term operation in practical engineering applications.

**4.** *Ensure a consistent writing style, as some sections are more technical, while others are relatively casual or less formal.*

**Response:**

We greatly appreciate the valuable feedback, which has significantly contributed to improving the quality of our paper. In response to this feedback, we have made the necessary revisions to ensure a more consistent, formal, and technical writing style throughout the manuscript and we have standardized the language to adopt a more formal tone in sections that were previously less formal.

**5.** *In the introduction and conclusion, emphasize the practical applications and real-world impact of the proposed model to align the technical work with practical significance.*

**Response:**

We acknowledge the importance of integrating technical details with practical applications. In response to your feedback, we have revised the "Introduction" and "Conclusion" sections of the manuscript to more explicitly highlight the practical applications of the BiStacking+TCN-GRU model.

**Introduction:** We have included a discussion on the role of electricity load forecasting in optimizing grid operations and power dispatching at the beginning of the Introduction. This addition underscores the critical importance of forecasting for maintaining grid stability and ensuring the efficient distribution of energy.

**End of the Introduction:** In the revised conclusion of the Introduction, we highlight the practical applications of the BiStacking+TCN-GRU model and explore its potential to enhance power load forecasting and improve grid stability.

**Conclusion:** In the Conclusion, we emphasize the practical applications of the proposed model, particularly its advantages in reducing the demand for computational resources and training time, which makes it more suitable for industrial deployment. Additionally, we highlight the model’s operational stability and maintainability, both of which are crucial for its large-scale implementation in real-world scenarios.

**6.** *Reorganize some of the experiment subsections to prevent overlapping descriptions, ensuring each section introduces new insights or findings.*

**Response:**

We fully agree with your suggestion and have removed the separate experimental section for the Stacking+TCN-GRU model, integrating its findings and conclusions into the comparative analysis of all models. Through this reorganization, we have presented the experimental results more coherently, ensuring that each section introduces new insights and findings, thereby avoiding redundancy. These revisions have enhanced the clarity and fluency of the manuscript, while more effectively highlighting the comparative performance of the proposed model relative to others.

**7.** *Include a comparison of model training times and computational costs for each model to evaluate trade-offs between performance and resource consumption.*

**Response:**

We acknowledge the importance of evaluating the trade-offs between model performance and resource consumption to assess the practical feasibility of different models. To address this, we conducted additional experiments comparing the training time and computational costs of each model. The relevant results are presented in Table 2, Section 4.3.3 of the revised manuscript. We believe that this addition enhances the manuscript by providing a more comprehensive evaluation of both the predictive performance and resource efficiency of the models.

**8.** *Justify the selection of TCN and GRU over other deep learning models or hybrid combinations, explaining why this combination was chosen.*

**Response:**

We have conducted a comparative analysis of various deep learning models, which is presented in Section 4.3.3 of the revised manuscript. The decision to combine TCN and GRU was made based on the following considerations:

**TCN’s Ability to Handle Complex Patterns:** As a variant of CNN, TCN effectively addresses the vanishing and exploding gradient problems commonly encountered in traditional RNNs. This enables TCN to capture long-range dependencies in sequential data while maintaining both stability and efficiency in processing complex patterns.

**GRU’s Efficiency in Sequential Learning:** In comparison to LSTM and BiLSTM, GRU simplifies the network architecture by reducing the number of gates and parameters, thereby improving training speed and making it more suitable for environments with limited computational resources.

**Comparison to Transformer Models:** Transformer-based models require significant computational resources, particularly during the training process. Additionally, the complexity of these models makes them computationally expensive and difficult to deploy in environments with limited resources.

By combining TCN with GRU, our model leverages the strengths of both CNN and RNN architectures while avoiding the computational overhead associated with more complex models, such as BiLSTM and Transformer-based networks. This hybrid approach achieves a balance between predictive accuracy, training efficiency, and computational resource consumption, making it particularly suitable for practical applications where computational resources and time are limited.

Thank you for your thorough review and valuable feedback on our manuscript. Your comments have greatly helped us identify the shortcomings of the paper and provided important directions for improvement. Your meticulous review not only enhanced the quality of the manuscript but also deepened our understanding of the research topic.

**Response to comments by Reviewer #2：**

**Comments：**

**1.** *How does the integration of BiStacking with TCN-GRU specifically improve predictive accuracy compared to traditional forecasting methods?*

**Response:**

To clarify that the improvement in predictive accuracy is primarily driven by the integration of BiStacking with TCN-GRU, we have provided additional details in Section 4.3.4, Experiment 3, of the revised manuscript.

The Tandem Base Learner in the BiStacking model enhances feature learning capabilities and effectively integrates the advantages of multiple Tandem Base Learners in the bidirectional selection layer. This improves the generalization ability of the model and enhances its ability to recognize complex data patterns.

TCN excel in capturing long-range dependencies and intricate temporal patterns. Compared to RNN, TCN effectively mitigate vanishing and exploding gradient issues, thereby substantially improving the model's capability to handle extended sequences.

The GRU employs a simplified gating mechanism that efficiently captures both short-term and long-term dependencies, while offering greater computational efficiency and faster training compared to other RNN architectures, such as LSTM.

By combining BiStacking with the TCN-GRU model, our approach efficiently integrates the prediction results of multiple Tandem Base Learners and fully leverages the advantages of ensemble learning. The robust sequential modeling capabilities of both TCN and GRU enable the BiStacking+TCN-GRU model to accurately capture the time-dependent and dynamic characteristics of the data, thereby significantly enhancing its ability to process complex time-series data, respond quickly to data fluctuations, and improve prediction accuracy and reliability in practical applications.

**2.** *I would highly suggest the authors to consolidate repetitive information, especially when discussing the results of multiple models, to avoid redundancy and enhance clarity.*

**Response:**

We fully agree with your recommendation, as it significantly enhances the clarity and coherence of the manuscript. In response, we have consolidated the results of Experiments 3 and 4 in Section 4.3.4 into a single, unified experiment. This revision reduces redundancy and streamlines the presentation of the results.

Additionally, we have removed redundant sections of the manuscript to enhance overall clarity and expression.

**3.** *The authors should try to elaborate more on how the model addresses potential issues related to overfitting, especially given the complexity introduced by the BiStacking approach.*

**Response:**

Your suggestions are invaluable in improving the manuscript. In response, we elaborate in Section 3.3 on the K-fold cross-validation and data normalization methods employed in the model to mitigate overfitting.

**K-fold Cross-validation:** By employing K-fold cross-validation, we ensure that the model is evaluated on multiple subsets of the data, thereby mitigating overfitting. This approach ensures that the model's performance is not overly reliant on any single training set and provides a more robust estimate of its generalization ability.

**Data Normalization:** We apply data normalization to standardize the input features, ensuring that they are on a comparable scale. This enhances the model's training stability and further reduces the risk of overfitting.

In summary, the BiStacking approach effectively addresses the potential for overfitting and enhances the robustness and generalization of the model through the integration of K-fold cross-validation and data normalization techniques.

**4.** *Although the graphs effectively illustrate the performance comparisons. I would recommend the authors to add error bars or statistical significance tests would strengthen the results.*

**Response:**

We fully agree with the suggestion to include error bars, and have added them to the graphs in Section 4.3.5 to more clearly illustrate the performance of the models. The inclusion of error bars enhances the presentation by demonstrating the stability and reliability of the results, particularly when comparing the performance of different models.

**5.** *How does this approach compare to other dynamic updating methods for rough sets or related models (e.g., fuzzy rough sets, probabilistic rough sets)?*

**Response:**

In our study, the BiStacking+TCN-GRU model combines the strengths of ensemble learning and deep learning. Unlike methods such as fuzzy rough sets and probabilistic rough sets, which primarily address uncertainty and fuzzy data, our model focuses on capturing the complex temporal patterns and both long- and short-term dependencies within electricity load data. As a TCN, it automatically learns multi-level features from time-series data, while the GRU effectively captures long-term dependencies through its gating mechanism. Therefore, our model excels in time-series modeling.

While methods like fuzzy rough sets and probabilistic rough sets are effective in handling noisy and incomplete data, they often require additional strategies to maintain high predictive performance when dealing with large volumes of time-series data. In contrast, our BiStacking+TCN-GRU model, leveraging the robustness of ensemble learning and the nonlinear modeling capabilities of deep learning, effectively handles noisy and incomplete data in electricity load forecasting, while maintaining high prediction accuracy.

To adapt to the complex and dynamic operating environment, this study designs a model normal update method that includes a correction mechanism, enabling rapid adaptation to changing operating conditions and load fluctuations when environmental conditions change significantly. Under extreme weather conditions, prediction model replicas specifically trained for such conditions is invoked to effectively address power load fluctuations caused by weather variations. Unlike approaches such as fuzzy rough sets and probabilistic rough sets, which rely on static rules and fixed data processing methods, our model achieves high prediction accuracy in dynamically changing load data through a flexible update strategy.

**6.** *Could this model be adapted for streaming data scenarios where objects are continuously added or removed?*

**Response:**

In order to adapt to the complex and ever-changing operating environment, this study combines offline training with online prediction methods to design a model update approach that includes a correction mechanism. This approach not only enables the model to adapt to changes in the operating environment, ensuring its stability and reliability in practical engineering applications, but also significantly reduces deployment costs and allows for timely updates using the most recent and historical data to maintain prediction accuracy. For streaming data scenarios, where objects are constantly added or removed, the current implementation mainly maintains prediction accuracy through regular updates.

The specific update method of the model is as follows: when the model is deployed, a prediction accuracy threshold is set according to the actual application requirements. When the model's prediction accuracy stabilizes within the threshold range, it is manually updated at regular intervals, retraining the model using the latest data and historical data within a specified time range. The correction mechanism is triggered by the prediction accuracy threshold. If the model's prediction accuracy falls below the threshold more than 10 times within a fixed interval, retraining is immediately triggered, until the accuracy returns to the threshold range. At the same time, the fixed interval is recalculated from the point at which the correction is triggered, ensuring the continuity and stability of the update strategy.

To provide the reader with a clear understanding of the model's update strategy, we have included relevant details in the conclusion.

**7.** *The Preliminaries section is generally helpful for establishing key definitions and concepts. Therefore, I would suggest the authors to consider moving some of the more technical details to methodlogical sections to improve readability for other researchers.*

**Response:**

We agree that the inclusion of overly technical details in the abstract can detract from its readability. In response, we have moved some of the technical details from the abstract to the methodological sections, where they can be discussed in more depth.

**8.** *The authors should mention the challenges and potential approaches for scaling this model to very large datasets or high-dimensional feature spaces.*

**Response:**

We appreciate the reviewer’s suggestion. In response, we discuss the challenges associated with scaling the model to large-scale datasets or high-dimensional feature spaces in the conclusion, and propose potential approaches to address these challenges:

**Increased Computational Resources**: As the dataset size and feature dimensionality increase, both the training time and computational resource requirements grow significantly. To mitigate this challenge, we propose distributed training, which allocates the computational load across multiple nodes or GPUs, thereby alleviating the pressure on a single node. Additionally, training with smaller batches can effectively reduce memory consumption and accelerate model convergence.

**Increased Memory Usage:** Larger datasets and higher-dimensional feature spaces lead to increased memory usage and computational complexity. To address this, we utilize the Pearson correlation coefficient to reduce the dimensionality of the input features, thereby minimizing the computational and memory burden while retaining the most relevant information about the load.

**Overfitting in High-Dimensional Data**: To prevent overfitting in high-dimensional data, we employ K-fold cross-validation to evaluate the model’s performance on different subsets of the data. Additionally, data normalization accelerates the training process and ensures that all features are on the same scale, thus effectively reducing the risk of overfitting and improving model stability.

We sincerely appreciate your constructive suggestions for our manuscript. Your recommendations have played a crucial role in improving the structure and expression of the paper. The specific revision suggestions you provided enabled us to effectively enhance the depth and breadth of the research, and also helped make the paper more aligned with academic standards.

Following the reviewers' valuable suggestions, we have carefully revised the entire manuscript. Last but not least, we would like to once again express our sincere gratitude to the reviewers and the editors for their insightful comments and suggestions, which have greatly contributed to enhancing the quality of our manuscript.

Yours Sincerely,

Jun Ma
