# Supplementary material for: A hybrid power load forecasting model using BiStacking and TCN-GRU
Source: PLoS One. 2025 Apr 28;20(4):e0321529. doi: 10.1371/journal.pone.0321529 (PMC12036927; doi:10.1371/journal.pone.0321529)
Supplement: Attachment — Submitted filename: Response_to_Reviewers_auresp_2.docx [file pone.0321529.s003.docx]

**Response to Reviewers**

**Dear Editors and** **Reviewers:**

We sincerely thank the Editors and reviewers for their insightful and constructive comments on our manuscript, *A Hybrid Power Load Forecasting Model Combining Ensemble Learning and Deep Learning: BiStacking+TCN-GRU*. We deeply appreciate the time and effort you dedicated to reviewing our work. Your valuable feedback has been instrumental in identifying areas for improvement and has provided essential guidance in refining the paper. Below is our detailed response to each of the reviewers' comments.

**Response to comments by Editors:**

Thank you for your valuable feedback. We have thoroughly reviewed the reference list and confirmed that all citations are complete and correct. Upon inspection, we found no retracted papers in the reference list.

**Response to comments by Reviewer #1:**

**Comments:**

**1.** *There are two subsection headings that include no overview about them, subsection 2.2 and subsection 4.3. Both the subsections don't provide any details about the section itself and move forward with it's subsequent subsections. I'd recommend the authors to fix this to improve readability and comprehension.*

**Response:**

Thank you for your valuable feedback. In response to your comment regarding the lack of an overview in subsections 2.2 and 4.3, we have added introductory paragraphs at the beginning of both sections to provide a clearer summary of their content. In subsection 2.2, we describe the base models and algorithms used in this study, with a particular focus on the TCN and Stacking models. In subsection 4.3, we outline the objectives of this section, which are to analyze and validate the performance of the proposed models through experimental results, and we also introduce the structure and progression of the subsequent subsections. We believe these modifications will improve the clarity and comprehensibility of these sections.

**2.** *Subsection 2.1 on "Data analysis and feature engineering" provides almost very little information about the topic. Although, it's a subsection, it should provide a more in-depth discussion on the Data analysis and feature engineering.*

**Response:**

Thank you for your valuable feedback. In response to your comment regarding the limited content in Subsection 2.1 on "Data analysis and feature engineering," we have expanded this section. We have highlighted the importance of data quality in time series forecasting and further discussed how effective data analysis and feature engineering are crucial in enhancing prediction accuracy and model efficiency. Additionally, we have included a discussion on how feature selection helps mitigate the risk of overfitting while also enhancing the model's training speed. These revisions provide a more detailed explanation of the significance of data analysis and feature engineering, and we believe they enhance the depth and clarity of this section.

**3.** *Inclusion of a literature review is needed. The research has conducted a comparison study, however, without a proper literature review among other works, it's very hard to judge the emphasize of the results.*

**Response:**

We have added a literature review to the paper, which will help readers gain a better understanding of our research contributions. Specifically, we have included the relevant literature review in the Introduction section, and we believe this addition will further enhance the quality of the paper.

**4.** *A proper section dedicated to further future works will enhance the discussion presented within the paper.*

**Response:**

Thank you for your valuable feedback. In response to your suggestion to add a dedicated section on future research directions, we have renamed the "Conclusion" section to "Conclusion and Future Research Directions" and included additional discussions on potential future research. Specifically, we emphasize that enhancing the model's ability to process large-scale datasets will be a key challenge in the context of smart grids. Moreover, we explore ways to improve the model’s ability to integrate multi-dimensional time series data, particularly with regard to factors such as weather, energy storage, and grid capacity, to improve the model's adaptability. Furthermore, we highlight the importance of broadening the model's prediction scope to better capture long-term trends and cyclical fluctuations. We believe these additions will significantly deepen the content of the paper and provide valuable guidance for future research.

Thank you for taking the time to review our paper and provide detailed feedback. Your suggestions have played a crucial role in improving the structure and content of the paper. In particular, your comments regarding the lack of overviews in subsections 2.2 and 4.3, as well as the brevity of the discussion in subsection 2.1, have helped us to better refine these sections. Furthermore, your feedback on the literature review and future research directions has added depth and clarity to the paper. We sincerely appreciate your thorough review and valuable suggestions.

**Response to comments by Reviewer #2:**

**Comments:**

**1.** *Title the Conclusion section "Conclusion and Future Research Directions" as it includes a few details about the future work as well.*

**Response:**

Thank you for your suggestion. We have updated the title of the Conclusion section to "Conclusion and Future Research Directions" and have included a discussion on future research directions in this section.

**2.** *Subsection 4.3 has no information about it's heading, it directly jumps into 4.3.1 and so on. This shows inconsistent flow of information. Add neccessary information about each heading instead of leaving it blank.*

**Response:**

Thank you for your valuable feedback. In response to your comment regarding the lack of an overview in Section 4.3, we have added an introductory paragraph at the beginning of this section to provide a clearer summary of its content.

In this revision, we outline the objectives of Section 4.3, aiming to analyze and validate the performance of the proposed model through experimental results. This section first introduces the experimental setup and data preprocessing, and then compares several deep learning models, demonstrating the performance advantages of the selected ensemble model and its potential for practical application. We believe these revisions will enhance the clarity of the section and improve the coherence of the information flow.

**3.** *Provide more information about the evaluation metrics. The information provided is not adaequte enough.*

**Response:**

In response to your suggestion to provide more information about the evaluation metrics, we have added a detailed explanation of the R-square, MAE, and RMSE metrics, including their principles and applications in power load forecasting. We have elaborated on how these metrics reflect model fitting, prediction error, and prediction accuracy, which helps clarify the model evaluation process. The additional content can be found in the "4.2 Evaluation Metrics" section of the manuscript. We believe that these additions will enhance the readability of the paper.

**4.** *Perhaps a related work section could improve the quality of the research paper and add more depth and weight to the research itself*

**Response:**

Thank you for your valuable suggestion. In response to your recommendation to include a related work section, we have made corresponding additions in Section 3.1 "Step 1: Stacking Part Construction." At the beginning of this section, we introduced research on the application of the Stacking model in forecasting tasks, providing a theoretical foundation for the introduction of the BiStacking model. Additionally, at the start of Section 3.2 "Step 2: Deep Learning Part Construction," we referenced relevant studies on TCN-LSTM and TCN-GRU to offer strong background support for the combination of BiStacking and TCN-GRU. Finally, at the beginning of Section 3.3 "Framework of the Forecasting Model," we included a brief description of the advantages of combining BiStacking and TCN-GRU. We believe these modifications enhance the academic depth of the paper and provide readers with a more comprehensive theoretical background and research foundation.

Thank you very much for reviewing our paper and providing valuable comments and suggestions. Your feedback has played an important role in further improving the content and structure of the paper. Your suggestions regarding the insufficient information in subsection 4.3, evaluation metrics, and the related work section have been very helpful, enabling us to enhance the clarity and depth of the paper. Once again, we sincerely appreciate your significant contribution to improving the quality of our work.

Following the reviewers' valuable suggestions, we have carefully revised the entire manuscript. Last but not least, we would like to once again express our sincere gratitude to the reviewers and the editors for their insightful comments and suggestions, which have greatly contributed to enhancing the quality of our manuscript.

Yours Sincerely,

Jun Ma
