# Supplementary material for: A hybrid power load forecasting model using BiStacking and TCN-GRU
Source: PLoS One. 2025 Apr 28;20(4):e0321529. doi: 10.1371/journal.pone.0321529 (PMC12036927; doi:10.1371/journal.pone.0321529)
Supplement: Attachment — Submitted filename: Response_to_Reviewers_auresp_3.docx [file pone.0321529.s004.docx]

**Response to Reviewers**

**Dear Editors and** **Reviewers:**

We sincerely thank the Editors and reviewers for their insightful and constructive comments on our manuscript, *A Hybrid Power Load Forecasting Model using BiStacking and TCN-GRU* We deeply appreciate the time and effort you dedicated to reviewing our work. Your valuable feedback has been instrumental in identifying areas for improvement and has provided essential guidance in refining the paper. Below is our detailed response to each of the reviewers' comments.

**Response to comments by Reviewer #3:**

Thank you for reviewing our manuscript once again and providing detailed and constructive feedback. Your suggestions are crucial for further enhancing the academic depth of our paper. We especially appreciate your insights on improving the literature review and your recommendations of key references, which have helped refine our discussion on hybrid prediction models.

**Comments:**

**1.** *After reviewing the revised manuscript, one area for improvement is the literature review. While it has been expanded, it would be beneficial to include more recent studies (past 2–3 years) on hybrid forecasting models combining ensemble and deep learning to enhance the study’s relevance and academic depth. Specifically, incorporating references such as 10.1007/s10489-021-02473-5 and 10.1007/s13042-024-02302-4 would strengthen the discussion.*

**Response:**

Thank you for your valuable feedback. We have incorporated and cited the two references you mentioned (10.1007/s10489-021-02473-5 and 10.1007/s13042-024-02302-4) in our manuscript. The innovative ideas presented in these papers are highly relevant to our research and provide significant insights for future research directions. The corresponding content has been marked in the **Introduction** section of the manuscript.

**Response to comments by Reviewer #4:**

Thank you for taking the time to review our manuscript and for providing profound and constructive feedback. Your insights have played a crucial role in enhancing the clarity, scientific rigor, and practical applicability of our paper. We are especially grateful for your suggestions regarding the integration of model components, computational efficiency, scalability, and the connection between experimental results and real-world power grid management. Furthermore, your recommendations on title optimization, literature updates, figure details, mathematical expressions, feature engineering visualization, evaluation metric explanations, and reference standardization have significantly helped us refine the paper’s presentation and academic rigor. Based on your feedback, we have carefully revised the manuscript and made detailed additions in relevant sections to further improve its quality and readability.

**Comments:**

**1.** *The manuscript needs to improve the clarity and depth of its explanations regarding the model's components and their integration. For instance, the rationale behind combining BiStacking and TCN-GRU should be elaborated, particularly how these methods complement each other in handling complex data patterns.*

**Response:**

BiStacking, as an ensemble learning method, focuses on mathematical modeling and optimization. By constructing multiple base learners and integrating their predictions, this method constructs multiple base learners and integrates their predictions to fully leverage the advantages of different base learners. Its core advantages lie in flexibility and efficiency, enabling different types of base learners to work collaboratively, thereby improving prediction accuracy. Additionally, BiStacking demonstrates strong generalization ability, making it suitable for various datasets while reducing the risk of overfitting associated with single models. Therefore, it is particularly effective in handling complex data. However, despite its advantages in dealing with complex datasets, BiStacking has limitations in capturing the long-term dependencies in time-series data.

TCN-GRU combines convolutional features with a gating mechanism to effectively capture long-term dependencies in time-series data. TCN extracts key features at different time scales through convolution operations, while GRU leverages its gating mechanism to effectively capture complex dynamics and nonlinear relationships in time-series data. TCN efficiently extracts global temporal features, whereas GRU further optimizes the transmission and retention of information. Therefore, in load forecasting tasks, TCN-GRU can effectively handle the dynamic characteristics of time-series data. In contrast, when using BiStacking alone, it may struggle to fully capture the long-term dependencies and nonlinear features inherent in time-series data.

When these two approaches are combined, BiStacking enhances the recognition and analysis of complex patterns by integrating multiple models, adjusting base model weights, optimizing the decision-making process, and improving the stability and interpretability of predictions. At the same time, TCN-GRU overcomes the limitations of single deep learning models by quickly responding to data fluctuations and enhancing the ability to process complex time-series data, thereby compensating for BiStacking’s weaknesses in temporal dependency modeling. The synergy between these two methods ultimately improves the accuracy and robustness of load forecasting.

The relevant content has been added to Section 4.3.4 BiStacking+TCN-GRU Experimental Results in the conclusion section of the paper.

**2.** *The manuscript should provide more details on the computational efficiency and scalability of the proposed model, especially when applied to datasets larger or more diverse than the one used in the study.*

**Response:**

Regarding computational efficiency and model scalability, the proposed BiStacking+TCN-GRU model has demonstrated strong computational efficiency in our experiments (see Section 4.3.3: Deep Learning Model Selection). By comparing the training time and prediction accuracy of state-of-the-art deep learning models under the same dataset, and comprehensively considering prediction time, accuracy, and memory consumption, the proposed BiStacking+TCN-GRU model effectively reduces computational costs while significantly improving prediction accuracy.

In terms of model scalability, the proposed BiStacking, as an ensemble learning method, can flexibly adapt to large-scale data by increasing the number of base learners or adjusting their types while optimizing based on data characteristics. To enhance the computational efficiency of deep learning models, future research could further explore incorporating an Attention mechanism into neural networks to dynamically allocate weights to temporal features, enabling more precise capture of key information. Additionally, integrating noise suppression and signal decomposition techniques in the data preprocessing stage could help uncover underlying patterns, providing stronger support for model optimization.

When applied to larger or more diverse datasets, the proposed model is well-suited for multi-feature load forecasting tasks and effectively selects relevant features, making it particularly applicable to datasets with high variability. To address the challenges posed by large-scale datasets, this study focuses on short-term load forecasting. Even with data collected hourly, the total number of samples within a year does not exceed 10,000. Within this data range, the proposed model meets forecasting requirements. To enhance the processing capability for large-scale datasets, future research could further integrate incremental learning and structural pruning techniques, along with optimization algorithms to adjust model parameters, thereby improving training efficiency on large-scale datasets.

We sincerely appreciate the reviewer’s insightful comments, which have been instrumental in refining our manuscript. The relevant additions have been incorporated and marked in the conclusion section to better highlight the contributions of this study.

**3.** *While the experimental results are comprehensive, the discussion does not sufficiently link these findings to practical applications in power grid management, which would strengthen the manuscript's contributions.*

**Response:**

Thank you for your valuable feedback. Your suggestions regarding the discussion of experimental results are crucial for improving the completeness of the paper and ensuring that the findings align more closely with real-world needs. Currently, the discussion lacks a strong connection between the experimental results and practical applications in power grid management. This is primarily because model deployment requires adjustments based on specific application scenarios and local conditions. Although this study does not directly involve practical applications, the experimental results were validated using real load data, ensuring the model's effectiveness in electricity load forecasting and demonstrating its potential for application in this field. Future research will further explore deployment strategies in real-world scenarios and optimize the model based on different application requirements. Once deployment conditions are clearly defined, the model will better serve the needs of power grid management, facilitating its practical implementation.

The relevant additions have been incorporated into the **Conclusion and Future Research** Directions section of the paper.

**4.** *The title should be streamlined for better readability; consider simplifying it while retaining key terms like "hybrid" and "forecasting."*

**Response:**

We sincerely appreciate the reviewer’s valuable comments. Based on your suggestion, we have revised the title from “A Hybrid Power Load Forecasting Model Combining Ensemble Learning and Deep Learning: BiStacking+TCN-GRU” to “A Hybrid Power Load Forecasting Model Using BiStacking and TCN-GRU”. We believe that this simplified title improves readability while retaining the essential keywords.

**5.** *The introduction should include more recent references to contextualize the novelty of the proposed approach.*

**Response:**

Thank you for your valuable suggestion. we have incorporated relevant studies from the past 2–3 years into the Introduction section to better highlight the novelty of the proposed method and to strengthen the academic background and practical relevance of the paper. The specific citations for the newly added references have been marked in the revised manuscript.

**6.** *Figures should include more detailed captions that explain the axes, key data points, and their relevance to the study.*

**Response:**

We sincerely appreciate the reviewer’s valuable suggestions. Based on your feedback, we have made the following modifications to the figures and charts. Specifically, we have added explanations for the x- and y-axes in the fitting plots and provided a detailed interpretation of the magnified sections in Section 4.3: Experimental Results Analysis. Additionally, for the bar chart summarizing the evaluation metrics, we have included additional explanations in the same section to clarify the results. Furthermore, we have emphasized key data points in the analysis and explored the relevance of the graphical results to the research objectives. These revisions aim to enhance readability and improve the accuracy of information transmission, ensuring that readers can better understand the figures. The specific modifications can be found in the annotated sections of the revised manuscript.

**7.** *The abstract contains redundant phrases and could be made more concise.*

**Response:**

Thank you for your valuable suggestion. Based on your suggestion, we have simplified the abstract by removing redundant expressions and emphasizing the core aspects of our research. We hope that this revision further enhances the clarity and readability of the paper.

**8.** *Certain mathematical equations lack clear variable definitions, which should be provided for better comprehension.*

**Response:**

Thank you for your valuable suggestion. The issue you raised is crucial, as providing detailed explanations of the variables in the equations helps readers better understand their meanings and roles within the formulas. We have thoroughly reviewed all equations and ensured that the definitions and descriptions of the relevant variables are complete and clear. Based on your suggestion, we have further refined the explanations of the equations to improve their comprehensibility and enhance the overall clarity of the paper.

**9.** *Section 2.1 on feature engineering should offer examples or visualizations to illustrate the impact of feature selection on model performance.*

**Response:**

Thank you for your valuable suggestion. To more clearly illustrate the impact of feature selection on model performance, we have added comparative charts of load forecasting results before and after feature engineering in Section 4.3.2. This visually demonstrates the improvement in prediction accuracy achieved through feature engineering, reinforcing the understanding of the importance of feature selection. Additionally, this section elaborates on the feature selection method in feature engineering, where low-correlation features are removed by calculating correlation coefficients, and experiments have been conducted to validate their impact on model prediction accuracy.

**10.** *A more detailed explanation of the evaluation metrics, particularly the trade-offs among RMSE, MAE, and R-squared, would benefit readers unfamiliar with these terms.*

**Response:**

Thank you for your valuable suggestion. In response to your suggestion, we have added more detailed explanations in the revised manuscript, particularly regarding the trade-offs between RMSE, MAE, and R-squared. We have clarified the definitions and characteristics of these evaluation metrics to help readers better understand their applications and significance. We believe that this revision will improve the readability of the paper, especially for readers who may not be familiar with these terms.

**11.** *The authors should include a justification for their choice of Panama's dataset and discuss how the results might generalize to other regions or timeframes.*

**Response:**

We sincerely appreciate the reviewer’s valuable comments. We selected the Panama power load dataset primarily because of its strong representativeness and broad applicability, making it particularly suitable for verifying the effectiveness of short-term load forecasting models. Additionally, this dataset is open-source, facilitating researchers in using and reproducing experiments, thereby promoting advancements in related research. This dataset spans a long period and exhibits significant seasonal fluctuations and complex load variation patterns, making it an ideal data source for testing the proposed hybrid forecasting model.

Regarding the generalizability of the model results, experimental findings indicate that the proposed model performs exceptionally well in power load forecasting tasks. We believe that for similar load forecasting tasks in different regions, the model can effectively adapt and generate predictions as long as historical load data from the target region is provided. For changing time periods, the manual update strategy used in the model accommodates different temporal requirements, as detailed in the Conclusion and Future Research Directions section. Additionally, the model efficiently handles multi-feature data, making it particularly suitable for complex load forecasting tasks.

Clarifying the rationale behind dataset selection provides strong support for the model’s application in multi-feature forecasting tasks. This addition not only enhances the rigor of the paper but also strengthens its practical relevance by demonstrating the model’s applicability across different regions and time periods. We sincerely appreciate the reviewer’s insightful suggestions, and the relevant additions have been incorporated into Section 4.1: Data Sources.

**12.** *The discussion section should further elaborate on the limitations of the proposed model and potential solutions.*

**Response:**

Thank you for your valuable suggestion. To address the limitations of the model and explore potential solutions, we have provided further elaboration:

1.Extreme Weather and Unexpected Events

Although the BiStacking+TCN-GRU model performs well in power load forecasting, its predictive accuracy may decline under extreme weather conditions or unexpected events. To address this issue, we propose a combination of offline training and online forecasting, along with a correction mechanism in the model’s regular update strategy. Additionally, a model replication strategy is introduced to enhance the model’s adaptability and stability under extreme weather conditions.

2.Handling of Anomalous Data

The model is sensitive to the quality of historical load data, and noise or missing values in the dataset may impact prediction accuracy. To improve the model’s robustness, we propose integrating outlier detection and noise filtering algorithms to clean anomalous data. Furthermore, we incorporate signal decomposition techniques to effectively separate noise components from trend components, thereby improving the model’s predictive accuracy and stability.

The relevant additions have been incorporated into the Conclusion and Future Research Directions section. We believe that these improvements provide a more comprehensive discussion of the model’s adaptability in complex and dynamic environments.

**13.** *The references section needs to be checked for consistency in formatting and completeness.*

**Response:**

Thank you for your valuable suggestion. The references in our manuscript follow the "Vancouver" style, as required by the Plos One journal. We have thoroughly reviewed and revised the reference formatting to ensure consistency and completeness in accordance with the journal’s guidelines.

Following the reviewers' valuable suggestions, we have carefully revised the entire manuscript. Last but not least, we would like to once again express our sincere gratitude to the reviewers and the editors for their insightful comments and suggestions, which have greatly contributed to enhancing the quality of our manuscript.

Yours Sincerely,

Jun Ma
